# The Effect of Acromioplasty or Bursectomy on the Results of Arthroscopic Repair of Full Thickness Rotator Cuff Tears: Does the Acromion Type Affect These Results?

**DOI:** 10.14744/SEMB.2021.12354

**Published:** 2021-12-29

**Authors:** Ozgun Karakus, Burak Gurer, Selcuk Kilic, Ahmet Sinan Sari

**Affiliations:** 1. Department of Orthopedics and Traumatology, Balikesir City Hospital, Balikesir, Turkey; 2. Department of Orthopedics and Traumatology, Omer Halisdemir University Hospital, Nigde, Turkey

**Keywords:** Acromion, bursitis, rotator cuff impingement, rotator cuff tears, shoulder pain

## Abstract

**Objectives:**

The aim of this study was to investigate the effect of subacromial decompression on the results of full thickness rotator cuff repair applied arthroscopically. Examination was also made of the effect of acromion type on the subacromial decompression procedure in patients applied with arthroscopic rotator cuff repair.

**Methods:**

The study included a total of 150 patients, comprising 102 (68%) females and 48 (32%) males with a full thickness rotator cuff tear repaired arthroscopically. The patients were separated into three groups of 50. Group A comprised those with acromioplasty and bursectomy applied additional to the repair. In Group B, only bursectomy was performed additional to the repair and in Group C, only rotator cuff repair was applied. Evaluation was made of the post-operative long-term pain and functional results.

**Results:**

The mean age of the cases was 65.63±9.22 years (range, 46–86 years). The affected side was right side in 95 (63.3%) cases and left side in 55 (36.7%). No statistically significant difference was determined between the groups according to the post-operative Constant Murley and ASES scores (p>0.05). In the paired comparisons, the post-operative VAS scores of Group C were higher than those of Groups A and B (p=0.018, p=0.029, p<0.05). No statistically significant difference was determined between Group A and Group B in respect of the post-operative VAS scores (p>0.05).

**Conclusion:**

In the arthroscopic repair of full thickness rotator cuff tears, neither acromioplasty, coracoacromial ligament loosening nor bursectomy were determined to have any positive effect on the results. Whatever the acromion type, there is no need for an additional subacromial decompression procedure after rotator cuff repair, in respect of pain and functional outcomes. Only acromial spurs should be gently removed paying attention to the coraco-acromial ligament.

Shoulder pain is described as the second most common musculoskeletal system disorder following lower back pain. Rotator cuff pathology and subacromial impingement are accepted as the leading causes of shoulder pain.^[1]^ Rotator cuff pathologies include partial or full thickness rotator cuff tears, subacromial bursitis, or rotator cuff tendinitis. Subacromial impingement syndrome has been accepted for many years as the most common reason for rotator cuff pathology. In 1949, Armstrong^[2]^ first suggested that the impingement of bursa and rotator cuff tendons below the acromion caused supraspinatus syndrome. Neer later reported that 95% of rotator cuff tears were due to mechanical impingement and treatment was successful with partial anterior acromioplasty.^[3,4]^

Arthroscopic acromioplasty is widely used in treatment of subacromial impingement. Despite application in cases not responding to conservative treatment, there are studies in literature reporting that there is no benefit of this procedure other than the additional costs.^[5,6]^

In general, there are two theories related to rotator cuff tear and subacromial impingement. These are the mechanical (extrinsic) theory and the degenerative (intrinsic) theory. The mechanical theory defined by Neer states that rotator cuff rupture occurs with the mechanical impingement of the coraco-acromial arch. Authors defending this theory have stated that acromioplasty is indispensible in rotator cuff repair. According to the degenerative theory, a tear forms associated with degeneration in the rotator cuff as a result of ageing and overuse of the shoulder. Those who advocate this theory suggest that acromioplasty is not necessary and the problem can be eliminated with bursectomy alone as all the symptoms and changes observed in the acromion are thought to have developed secondary to degenerative tendinopathy.

Although there are studies and clinical data supporting both theories, no consensus has been reached as yet.^[7,8]^

Recent studies have questioned the role of subacromial impingement in rotator cuff tears. There are studies showing that the effect of partial acromioplasty applied in cases of rotator cuff pathology has not changed the results as much as expected.^[9-11]^

There are also studies which have stated coracoacromial ligament damage as the reason for deltoid detachment. In a cadaver study by Green et al., it was shown that a 4mm bone resection made from the acromial surface caused 56% loosening in the attachment site of the deltoid and a 5.5 mm bone resection caused 77% loosening in the deltoid attachment.^[12]^

In contrast, there are also studies that recommend bursectomy only in the treatment of rotator cuff disease and impingement syndrome. These studies suggest that an inflamed and thickened bursa is a major source of pain and creates impingement; therefore, bursectomy would be extremely beneficial.^[13,14]^ In studies related to the shape of the acromion or acromial spurs, a relationship has been determined between these pathologies and rotator cuff tears, but the cause and effect relationship has not been fully revealed.^[15]^

Effective results have been reported of rotator cuff repair applied without acromioplasty or coracoacromial ligament loosening. There are also studies stating that acromioplasty applied together with coracoacromial ligament loosening could cause an increase in glenohumeral instability.^[16,17]^ In contrast, other studies in literature have shown excellent results of rotator cuff repair applied together with subacromial decompression.^[18]^

Therefore, no consensus has yet been reached and the aim of this study was to investigate the effect of subacromial decompression in full thickness rotator cuff repair performed arthroscopically, that is, the effect on the results of anterior-inferior acromioplasty, coracoacromial ligament loosening and subacromial bursectomy. A different aspect of this study is that evaluation was made separately of the effect on the results of acromioplasty, coracoacromial ligament release and subacromial bursectomy. In addition, it was examined whether the subacromial decompression procedure showed any difference according to the acromion type in patients applied with arthroscopic rotator cuff repair, or whether evaluation should be made independently of acromion type.

## Methods

This study has been performed by Niğde Ömer Halisdemir University Hospital Department of Orthopedics and Traumatology, with the Niğde Ömer Halisdemir University Local Ethics Committee decision dated 10/12/2019 No: 57.

The study included a total of 150 patients applied with arthroscopic full thickness rotator cuff repair, comprising 102 (68%) females and 48 (32%) males with a mean age of 65.63 ± 9.22 years (range, 46–86 years). The affected side was right side in 95 (63.3%) cases and left side in 55 (36.7%). Patients were excluded if they had shoulder instability, glenohumeral joint degenerative arthritis, acromioclavicular pathology, tuberculum fracture, a history of shoulder surgery because of fracture, or if arthroscopic repair was applied again because of re-rupture following primary repair.

All the patients had a full thickness, crescent type tear. In all cases, arthroscopic examination was made of the glenohumeral joint, and no additional pathology was determined on the long biceps head. The patients were separated into three groups of 50. Group A comprised those applied with arthroscopic rotator cuff repair, bursectomy, coracoacromial ligament loosening and antero-inferior acromioplasty. In Group B, arthroscopic rotator cuff repair was applied with bursectomy only and no acromioplasty. In Group C, only rotator cuff repair was applied with no additional subacromial decompression procedure. In Groups B and C, any acromial spurs determined during arthroscopy were lightly corrected with a burr, but patients with type III acromion could not be converted to types I and II acromion.

With pre-operative magnetic resonance imaging (MRI) and X-ray examinations of all patients, the subacromial area, acromion type, (according to Bigliani on MRI scans: type I- flat, type II-curved, and type III-hooked), and full rotator cuff pathology were determined and recorded. All patients were evaluated preoperatively with measurement of range of motion, the Constant Murley (CM) Score, the American Shoulder and Elbow Surgeons (ASES) Score, and Visual Analog Score (VAS) for pain, and the results were recorded.

The mean follow-up period was 36.2 months (range, 26–51 months). At the final follow-up examination, all the patients were again evaluated again with the CM, ASES, and VAS scores, and the acromion type was measured again with post-operative MRI. These values were compared with the pre-operative values. All the tests at the final follow-up examination were applied by two orthopedic surgeons. Differences between the groups were examined in respect of the shoulder function and pain scores.

### Surgical Technique

Prophylaxis of 1000 g cefazol was administered, then under general anesthesia the patients were placed in the beach-chair position. Following the necessary draping and staining procedures, a posterior portal was opened and arthroscopic examination of the glenohumeral joint was made. Then entering the subacromial space, first a smooth opening and visualization was obtained with radiofrequency and a shaver, then the rotator cuff tear was visualized. By determining the footprint, the rotator cuff was compressed with a 4.5 mm titanium anchor (Arthrex®, Smith and Nephew®) using the lateral portal. With the tension band method, double row repair was made using a 4.5 mm pushLock anchor. In Group A patients, following bursectomy with the aid of radiofrequency and a shaver, coracoacromial ligament loosening and antero-inferior acromioplasty using a 4 mm burr were also applied. To the patients in Group B, only bursectomy was applied.

The same post-operative follow-up protocol was applied to all the patients in all the groups. Passive shoulder movements were started on postoperative day 1. For 6 weeks, a shoulder-arm sling supporting abduction was worn and active shoulder movement was restricted for 6 weeks. The sling was removed after 6 weeks, and a physical therapy program was applied in respect of active shoulder movements for 2 months. No complications were observed in any patient throughout the post-operative follow-up period.

### Statistical Analysis

Data obtained in the study were analyzed statistically using NCSS software (Number Cruncher Statistical System, 2007, Kaysville, Utah, USA). When evaluating the data descriptive statistical methods were used and results were stated as mean±standard deviation (SD), median, minimum and maximum values, number (n), and percentage (%). Conformity of quantitative data to normal distribution was assessed with the Kolmogorov–Smirnov test, the Shapiro–Wilk test and graphically. In the comparison of three or more groups of data showing normal distribution, the one-way ANOVA test was used, and if not conforming to normal distribution, the Kruskal–Wallis test. In paired comparisons the Bonferroni-Dunn test was applied. In the comparison of qualitative data, the Pearson Chi-square test was used. In the within group comparisons, the Paired Samples t-test was applied to parameters showing normal distribution and the Wilcoxon Signed-Rank test to parameters not showing normal distribution. The Homogeneity test was applied in the evaluation of preoperative and post-operative acromion types. A value of p<0.05 was accepted as statistically significant.

## Results

Evaluation was made of a total of 150 patients applied with arthroscopic full thickness rotator cuff repair, comprising 102 (68%) females and 48 (32%) males with a mean age of 65.63±9.22 years (range, 46–86 years). The affected side was right side in 95 (63.3%) cases and left side in 55 (36.7%).

No statistically significant difference was determined between the groups in respect of age and gender distribution or the operated side (p>0.05) (Table 1).

**Table 1.** Demographic characteristics according to the groups

	**Total (n=150)**	**Group A (n=50)**	**Group B (n=50)**	**Group C (n=50)**	**P**
Age (years)					
Min–Max (Median)	46–86 (65.5)	48–81 (67)	46–81 (65)	46–86 (61)	0.786^a^
Mean±SD	65.63±9.22	66.36±8.81	65.40±9.60	65.14±9.36	
Gender; n (%)					
Female	102 (68.0)	30 (60.0)	36 (72.0)	36 (72.0)	0.332^b^
Male	48 (32.0)	20 (40.0)	14 (28.0)	14 (28.0)	
Side; n (%)					
Right	95 (63.3)	35 (70.0)	25 (50.0)	35 (70.0)	0.057^b^
Left	55 (36.7)	15 (30.0)	25 (50.0)	15 (30.0)	

^a^One-way ANOVA test; ^b^Pearson Chi-square test.

In the evaluation of the CM scores, no statistically significant difference was determined between the groups in respect of the pre-operative and post-operative CM scores (p>0.05). In Groups A, B, and C, a statistically significant increase was determined in the postoperative CM scores compared to the preoperative values (p=0.001, p<0.05, for all). No statistically significant difference was determined between the groups in respect of the change in the CM score from pre-operative to postoperative (p>0.05) (Table 2).

**Table 2.** Evaluation of CM, ASES, VAS scores, and acromion types according to the groups

	**Total (n=150)**	**Group A (n=50)**	**Group B (n=50)**	**Group C (n=50)**	**P**
CM score					
Pre-operative					
Min–Max (Median)	35–48 (39)	35–48 (39)	36–48 (39)	36–48 (39)	0.875^a^
Mean±SD	39.55±2.32	39.68±2.60	39.54±2.30	39.44±2.07	
Post-operative					
Min-Max (Median)	72–89 (80)	76–89 (80)	72–87 (80)	72–89 (82)	0.470^a^
Mean±SD	81.12±2.73	81.00±2.49	80.86±2.61	81.50±3.06	
Pd	0.001**	0.001**	0.001**		
Pre-operative-post-operative difference					
Min-Max (Median)	32–50 (42)	32–49(41.5)	32–49 (42)	32–50 (42)	0.443^a^
Mean±SD	41.57±3.33	41.32±3.33	41.32±3.15	42.06±3.52	
ASES score					
Pre-operative					
Min-Max (Median)	20–32 (24)	20–32 (23)	20–32 (23)	21–32 (25)	0.975^a^
Mean±SD	24.07±2.83	24.10±2.83	24.00±2.98	24.12±2.72	
Post-operative					
Min–Max (Median)	68–93 (80)	70–93 (80)	68–93 (80)	70–93 (80)	0.881^a^
Mean±SD	79.89±4.14	79.90±3.68	79.68±3.94	80.10±4.80	
Pd	0.001**	0.001**	0.001**		
Pre-operative-post-operative difference	
Min–Max (Median)	41–72 (56)	48–71 (56)	46–72 (56)	41–72 (56)	0.955^a^
Mean±SD	55.82±4.94	55.80±4.25	55.68±4.64	55.98±5.88	
VAS score					
Pre-operative					
Min–Max (Median)	4–10 (8)	4–9 (8)	4–9 (8)	5–10 (8)	0.332^c^
Mean±SD	7.51±1.19	7.56±1.13	7.32±1.24	7.66±1.19	
Post-operative					
Min–Max (Median)	1–7 (2)	1–4 (1)	1–4 (1)	1–7 (2)	0.032*^c^
Mean±SD	1.79±1.11	1.56±0.73	1.60±0.78	2.22±1.52	
Pe	0.001**	0.001**	0.001**		
Pre-operative-post-operative difference					
Min–Max (Median)	−9–−2 (−6)	−8–−2 (−6)	−8–−2 (−6)	−9–−2 (−6)	0.175^c^
mean±SD	−5.72±1.49	−6.00±1.34	−5.72±1.50	−5.44±1.61	
Acromion type					
n (%)					
Pre-operative					
Type 1	59 (39.3)	20 (40.0)	22 (44.0)	17 (34.0)	0.771^b^
Type 2	63 (42.0)	19 (38.0)	20 (40.0)	24 (48.0)	
Type 3	28 (18.7)	11 (22.0)	8 (16.0)	9 (18.0)	
Post-operative					
Type 1	61 (40.7)	24 (48.0)	20 (40.0)	17 (34.0)	0.101^b^
Type 2	71 (47.3)	25 (50.0)	22 (44.0)	24 (48.0)	
Type 3	18 (12.0)	1 (2.0)	8 (16.0)	9 (18.0)	

^a^One-way ANOVA test; ^b^Pearson Chi-square test; *p<0.05; **p<0.01; ^c^Kruskal Wallis test; ^d^Paired Samples t-test; ^e^Wilcoxon Signed-Ranks test. ASES: American shoulder and elbow surgeons score; CM: Constant Murley score; VAS: Visual analog score.

In the evaluation of the ASES scores, no statistically significant difference was determined between the groups in respect of the preoperative and postoperative ASES scores (p>0.05). In Groups A, B, and C, a statistically significant increase was determined in the post-operative ASES scores compared to the pre-operative values (p=0.001, p<0.05, for all). No statistically significant difference was determined between the groups in respect of the change in the ASES score from pre-operative to postoperative (p>0.05) (Table 2).

In the evaluation of the VAS scores, no statistically significant difference was determined between the groups in respect of the pre-operative and post-operative VAS scores (p>0.05). A statistically significant increase was determined between the groups in respect of the postoperative VAS scores (p=0.032, p<0.05). As a result of the paired comparisons, the post-operative VAS scores of Group C were determined to be statistically significantly higher than those of Groups A and B (p=0.018, p=0.029; p<0.05). No statistically significant difference was determined between the postoperative VAS scores of Group A and Group B (p>0.05). In Groups A, B, and C, the decrease in the post-operative VAS scores compared to the preoperative values was determined to be statistically significant (p=0.001; p<0.05 for all). No statistically significant difference was determined between the groups in respect of the change in the VAS score from preoperative to postoperative (p>0.05) (Table 2).

In the evaluation of the post-operative VAS scores according to acromion type;

In Group A, when the post-operative VAS scores were evaluated according to postoperative acromion type, the VAS score of cases with type I acromion was determined to be statistically significantly higher than that of cases with type II acromion (p<0.01).

In Group B, a statistically significant difference was determined in postoperative VAS scores according to acromion type (p=0.011; p<0.05). The VAS scores of cases with type I acromion were determined to be statistically significantly higher than those of cases with type II acromion (p=0.09; p<0.01). No statistically significant difference was determined between the VAS scores of other types (p>0.05).

In Group C, a statistically significant difference was determined in post-operative VAS scores according to acromion type (p=0.011; p<0.05). When significance was examined with the Dunn test, the post-operative VAS scores of cases with type III acromion were determined to be statistically significantly higher than those of cases with type I and type II acromion (p=0.021; p=0.001). No statistically significant difference was determined between type I and type II (p>0.05) (Table 3).

**Table 3.** Evaluation of post-operative VAS scores according to post-operative acromion types

**Post-operative Acromion type**	**N**	**Post-operative VAS Score**	**P**
		**Mean**	**SD**	**Min-Max (median)**	
Group A					
Type 1	24	1.88	0.80	1–4 (2)	
Type 2	25	1.28	0.54	1–3 (1)	0.003**
Type 3	1	1.00	.	1–1 (1)	
Group B					
Type 1	20	1.95	0.89	1–4 (2)	
Type 2	22	1.23	0.43	1–2 (1)	0.011*
Type 3	8	1.75	0.89	1–3 (1.5)	
Total	50	1.60	0.78	1–4 (1)	
Group C					
Type 1	17	1.94	0.75	1–3 (2)	
Type 2	24	1.58	0.65	1–3 (1.5)	0.002**
Type 3	9	4.44	2.19	1–7 (5)	

^c^Kruskal–Wallis test; ^f^Mann–Whitney U test. VAS: Visual analog score.

In Group A, the change in acromion type from preoperative to post-operative was found to be statistically significant (p=0.016; p<0.05).

Preoperatively, acromion type was type I in 20 cases, type II in 19 cases, and type III in 11 cases. Postoperatively, acromion type was type I in 24 cases, type II in 25 cases, and type III in 1 case.

Of the 20 cases with pre-operative type I acromion, 14 remained as type I postoperatively, and 6 changed to type II. Of the 19 cases with type II acromion preoperatively, 13 remained as type II postoperatively, and 6 changed to type I. Of the 11 cases with pre-operative type III acromion, 1 remained as type III postoperatively, 4 changed to type I and 6 to type II.

In Group B, the change in acromion type from pre-operative to post-operative was not found to be statistically significant (p=0.157; p>0.05).

Preoperatively, acromion type was type I in 22 cases, type II in 20 cases, and type III in 8 cases. Postoperatively, acromion type was type I in 20 cases, type II in 22 cases, and type III in 8 cases.

Of the 22 cases with pre-operative type I acromion, 20 remained as type I postoperatively, and 2 changed to type II. All the 20 cases with type II acromion preoperatively remained as type II postoperatively. All the eight cases with pre-operative type III acromion remained as type III postoperatively.

In Group C, the change in acromion type from pre-operative to post-operative was not found to be statistically significant (p=1.000; p>0.05).

All the 17 cases with pre-operative type I acromion remained as type I postoperatively. All the 24 cases with type II acromion preoperatively remained as type II postoperatively. All the nine cases with pre-operative type III acromion remained as type III postoperatively (Table 4).

**Table 4.** Within group evaluations of pre-operative and post-operative acromion types

**Groups**	**Post-operative Acromion Type**	**Pre-operative Acromion Type**				**P**
		**Type 1**	**Type 2**	**Type 3**	**Total**	
		**n (%)**	**n (%)**	**n (%)**	**n (%)**
Group A						
	Type 1	14 (28.0)	6 (12.0)	4 (8.0)	24 (48.0)	0.016*
	Type 2	6 (12.0)	13 (26.0)	6 (12.0)	25 (50.0)
	Type 3	0 (0)	0 (0)	1 (2.0)	1 (20.0)
	Total	20 (40.0)	19 (38.0)	11 (22.0)	50 (100.0)
Group B						
	Type 1	20 (40.0)	0 (0)	0 (0)	20 (40.0)	0.157
	Type 2	2 (4.0)	20 (40.0)	0 (0)	22 (44.0)
	Type 3	0 (0)	0 (0)	8 (16.0)	8 (16.0)
	Total	22 (44.0)	20 (40.0)	8 (16.0)	50 (100.0)
Group C						
	Type 1	17 (34.0)	0 (0)	0 (0)	17 (34.0)	1.000
	Type 2	0 (0)	24 (48.0)	0 (0)	24 (48.0)	
	Type 3	0 (0)	0 (0)	9 (18.0)	9 (18.0)	
	Total	17 (34.0)	24 (48.0)	9 (18.0)	50 (100.0)	

^f^Marginal Homogeneity test; *p<0.05.

## Discussion

For many years, subacromial impingement syndrome has been shown to be the reason for rotator cuff pathology. Although there are studies showing that acromial spurs and acromion types are related to rotator cuff pathology, this has not been fully proven. There are also studies that have stated that rather than the type of acromion, other factors cause the impingement in the formation of rotator cuff pathology.^[19-22]^

Studies in literature by authors supporting the extrinsic theory have stated that routine application of acromioplasty both improves the arthroscopic visualization and induces healing of the bleeding from the bone which occurs with the acromioplasty in the subacromial space.^[23,24]^

In contrast, those who support the intrinsic theory have suggested that preserving the coracoacromial ligament in respect of glenohumeral stability both reduces costs and shortens operating time and there is no need for acromioplasty following successful rotator cuff repair.^[25-27]^

No consensus has been reached in literature as yet. The aim of the current study was to show whether or not there was any benefit of subacromial decompression according to acromion type in patients applied with rotator cuff repair. Evaluation was made of 150 patients with the CM score, the ASES score, and the VAS score for pain.

When evaluations were made in respect of the CM score, there was a significant difference between the pre-operative and post-operative scores of all the groups (p=0.001, p<0.05). However, there was no difference between the post-operative scores of the three groups (p>0.05). This demonstrated that all the groups benefitted from surgery. The application of acromioplasty or bursectomy without acromioplasty or not making any additional procedure other than rotator cuff repair was seen not to make any difference in respect of the CM score after rotator cuff repair.

There was a significant difference between the pre-operative and post-operative ASES scores of all the groups (p=0.001, p<0.05). However, there was no difference between the post-operative ASES scores of the three groups (p>0.05). This demonstrated that all the groups benefitted from surgery. Thus the application of acromioplasty, bursectomy, or no subacromial bursectomy procedure following rotator cuff repair was not seen to make any significant difference in respect of the ASES score. There are similar results in literature.^[28,29]^

However, when the VAS scores were evaluated, a significant difference was determined between the groups in respect of the post-operative VAS scores (p=0.032, p<0.05). The point of interest here is that there was no statistically significant difference between the group applied with acromioplasty additional to the rotator cuff repair (Group A) and the group where only bursectomy was performed after the repair (Group B) (p>0.05). Irrespective of acromion type, acromioplasty or bursectomy applied in addition to rotator cuff repair made no difference in respect of the VAS scores. However, the post-operative VAS scores of the group not applied with any subacromial decompression procedure in addition to rotator cuff repair (Group C) were significantly higher than those of Groups A and B (p=0.018, p=0.029; p<0.05). When Group C was examined, the patients with high post-operative VAS scores were seen to be those with type III acromion (p=0.021, p=0.001). No significant difference was seen between the cases with type I and type II acromion in respect of post-operative VAS scores (p>0.05) (Table 2).

In Group A, when the VAS scores were evaluated according to acromion type, the VAS scores of cases with type I acromion were determined to be significantly higher than those of cases with type II acromion (p<0.01).

A significant difference was determined in Group B when the postoperative VAS scores were evaluated according to acromion type (p=0.011, p<0.05). The VAS scores of the cases with type I acromion were determined to be significantly higher than those of cases with type II acromion (p=0.009, p<0.01). No significant difference was determined between the other acromion types in this group in respect of post-operative VAS scores (p>0.05) (Table 3).

Finally, it was clear that acromion type showed no correlation with the VAS score. While the VAS scores of cases with type I acromion in Groups A and B were higher than those of the cases with type II, in Group C the highest VAS scores were in cases with type III acromion. Similar results have been reported in some previous studies.^[30]^

Although this shows that antero-inferior acromioplasty or bursectomy applied without being aggressive after rotator cuff repair in patients with type III acromion provided a benefit in respect of the pain score, there was still seen to be no correlation between VAS score and acromion type. The previous studies have shown results that the shoulder scores of cases with type III acromion were worse than those of type I acromion.^[31]^

As no consensus has been reached on this subject in literature, the cause and effect relationship has not been revealed. In the current study, type III acromion only affected the postoperative VAS score, and did not seem to affect the ASES and CM scores. Furthermore, there was no correlation of clinical significance between the postoperative acromion type and VAS score. This could be attributed to the VAS score not being a functional score, but an isolated pain score, and as it is a highly subjective scoring of pain, can vary from person to person.

However, the decrease in VAS scores from pre-operative to postoperative was found to be statistically significant in all the groups (p=0.001, p<0.05). Thus, it can be said that all the groups benefitted from the surgery, and this benefit seems to be clearly related to the rotator cuff repair.

Very interesting results emerged from evaluating the pre-operative and post-operative acromion types of the patients. In Group A where acromioplasty was applied in addition to rotator cuff repair, of the 20 cases with type I acromion preoperatively, 14 cases remained as type I postoperatively and 6 cases changed to type II. Of the 19 cases with preoperative type II acromion, 13 remained as type II postoperatively and 6 changed to type I. Of the 11 cases with preoperative type III acromion, only 1 case remained type III postoperatively, 4 changed to type I and 6 to type II (Table 4).

When performing acromioplasty in Group A, the target was to convert type II and type III acromions to type I. The target in type I acromions, because of the narrowed subacromial space, was only to remove acromial spurs. In the measurements taken after approximately 3 years, a third of the type I acromions had changed to type II, and the majority of type II acromions remained as type II. By applying acromioplasty to convert to type I acromion, only 1 of the type III acromions remained as type III and the majority changed to type II. Together with ageing, there seems to be the formation of new bone in the site of the resected bone piece. This could show that within years of aggressively applied acromioplasty, the formation of new bone is induced and this does not have a very positive effect on functional scores. There are studies in the literature showing that the acromion type changed within years after acromioplasty.^[30]^

In Group B, where only bursectomy was applied in addition to rotator cuff repair, of the 22 cases with pre-operative type I acromion, 20 cases remained as type I postoperatively and 2 changed to type II. All the 20 pre-operative type II cases remained as type II postoperatively, and all the eight pre-operative type III cases also remained as type III postoperatively.

In Group C, where only rotator cuff repair was applied, all the 17 pre-operative type I acromion cases remained as type I postoperatively. The 24 pre-operative acromion type II cases all remained as type II postoperatively and the 9 pre-operative type III cases also all remained as type III postoperatively.

The acromion morphology is known to change with age and an increase is seen in type III acromion together with ageing. However, the majority of patients with type III acromion are asymptomatic and the presence of type III acromion alone is not sufficient for the formation of impingement syndrome.^[32]^ According to the results of the current study, following intervention to the subacromial area such as acromioplasty, there clearly seems to be a change occurring in the acromion type which is induced by new bone formation.

In conclusion, the change of acromion type is not of great importance in a clinical sense, and over time, the bone which has been taken may form again. No subacromial decompression procedure applied in addition to rotator cuff repair seems to have a positive effect on results. In the presence of acromial spurs only, gentle spur excision can be performed, but the target should never be to change acromion type.

When Group C, where rotator cuff repair only was performed, was compared with the other groups, no significant difference was determined in all the functional scores. Only when evaluations were made in respect of the VAS scores, the scores of the patients with type III acromion were determined as high compared to the other groups and the cases with type I and type II acromion in Group C. However, as can be seen in the same table (Table 3), in the evaluations of post-operative VAS scores according to postoperative acromion types in Groups A and B, the VAS scores of cases with type I acromion were determined as statistically significantly higher than those of cases with type II acromion (p<0.01).

No significant difference was found between the groups in respect of the post-operative CM and ASES scores, but there were differences in the VAS scores. As the CM and ASES scores provide a more detailed evaluation in respect of both function and pain, they are thought to be more accurate results. That no correlation was seen between VAS score and acromion type can be explained by it being a completely subjective evaluation.^[30]^

Nevertheless, there were some limitations to the study, primarily the low numbers of the groups, sizes of acromial spurs, surgery time, additional chronic disease, post-operative adaptation to rehabilitation, size of the tear, and U- and L-shaped full thickness rotator cuff tear repair results were not evaluated.

## Conclusion

The results of this study showed that neither acromioplasty, nor coracoacromial ligament release, nor bursectomy were determined to have a positive effect on the results of the arthroscopic repair of full thickness rotator cuff tears. Following rotator cuff repair, whatever the acromion type, there is no need for the application of any additional subacromial decompression procedure in respect of pain and functional outcome. When there are only acromial spurs, these should be minimal removed, paying attention to the coracoacromial ligament. The aim in acromioplasty should never be to convert type II and type III acromions to type I. The aggressive application of acromioplasty was seen to induce new bone formation over time.

### Disclosures

**Ethics Committee Approval:** This study has been performed by Niğde Ömer Halisdemir University Hospital Department of Orthopedics and Traumatology, with the Niğde Ömer Halisdemir University Local Ethics Committee decision dated 10/12/2019 No: 57.

**Peer-review:** Externally peer-reviewed.

**Conflict of Interest:** None declared.

**Authorship Contributions:** Concept – O.K.; Design – O.K., A.S.S.; Supervision – O.K., B.G.; Materials – O.K., A.S.S.; Data collection &/or processing – O.K., B.G.; Analysis and/or interpretation – S.K., B.G.; Literature search – O.K., A.S.S.; Writing – O.K.; Critical review – S.K., B.G.
